# A PET-CT study on the specificity of acupoints through acupuncture treatment in migraine patients

**DOI:** 10.1186/1472-6882-12-123

**Published:** 2012-08-15

**Authors:** Jie Yang, Fang Zeng, Yue Feng, Li Fang, Wei Qin, Xuguang Liu, Wenzhong Song, Hongjun Xie, Ji Chen, Fanrong Liang

**Affiliations:** 1 Acupuncture and Tuina School, Chengdu University of Traditional Chinese Medicine, Chengdu, 610075, China; 2 Life Science Research Center, School of Life Science and Technology, Xidian University, Xi’an, Shaanxi, 710071, China; 3 PET-CT center, Sichuan Provincial People’s Hospital, Chengdu, 610072, China; 4 Acupuncture Department, The third affiliated hospital of zhejiang university of TCM, hangzhou, 310005, China

**Keywords:** Acupuncture, PEC-CT, Migraine

## Abstract

**Background:**

In the field of acupuncture research, the topic of acupoint specificity has received increasing attention, but no unified conclusion has been reached on whether or not acupoint specificity exists. Furthermore, the majority of previous acupuncture neuroimaging studies have been performed using healthy subjects. In this study, patients with migraine were used to investigate acupoint specificity.

**Methods:**

Thirty patients with migraine were enrolled and randomized into three groups: Traditional Acupuncture Group (TAG), Control Acupuncture Group (CAG), and Migraine Group (MG). The TAG was treated by acupuncture stimulation at Waiguan (TE5), Yang Lingquan (GB34), and Fengchi (GB20). The CAG was treated at Touwei (ST8), Pianli (LI6), and Zusanli (ST36). The MG received no treatment. Positron emission tomography with computed tomography (PET-CT) was used to test for differences in brain activation between the TAG and CAG versus MG, respectively.

**Results:**

Traditional acupuncture treatment was more effective for pain reduction than control acupuncture treatment. The TAG showed higher brain metabolism than the MG in the middle temporal cortex (MTC), orbital frontal cortex (OFC), insula, middle frontal gyrus, angular gyrus, post-cingulate cortex (PCC), the precuneus, and the middle cingulate cortex (MCC). Metabolism decreased in the parahippocampus, hippocampus, fusiform gyrus, postcentral gyrus, and cerebellum in the TAG compared with the MG. In the CAG, metabolism increased compared with the MG in the MTC, supratemporal gyrus, supramarginal gyrus, and MCC, whereas metabolism decreased in the cerebellum.

**Conclusions:**

Acupuncture stimulation of different points on similar body regions in migraine patients reduced pain and induced different levels of cerebral glucose metabolism in pain-related brain regions. These findings may support the functional specificity of migraine- treatment-related acupoint.

**Trial registration:**

The number of our clinical trial registration is: ChiCTR-TRC-11001813, and the protocol and inclusion criteria have already been registered as ChiCTR-TRC-11001813.

## Background

Acupuncture, an important part of Traditional Chinese Medicine (TCM), has been accepted as an ancient therapeutic modality in Eastern medicine. However, little is known about the neural mechanisms of acupuncture. In the past decade, positron emission tomography (PET) and functional magnetic resonance imaging (fMRI) techniques have provided a means to study effects of acupuncture on the brain, and to elucidate the mechanisms of action in acupuncture treatment of diseases [1-4].

In acupuncture research, the topic of acupoint specificity has received increasing attention. Significant neuroimaging evidence of acupoint specificity of the vision-related acupoints was provided by Cho *et al*[5]. Other studies have also focused on the specificity of acupoints, including visual and auditory acupoints [6-9], but no consensus has yet been reached on the existence of acupoint specificity. The majority of previous acupuncture neuroimaging studies have used healthy subjects. To our knowledge, only a few neuroimaging studies have reported on the response to acupuncture in patients with disorders [10-12].

Migraine is one of the indications for acupuncture therapy. Randomized controlled trials (RCTs) have shown that acupuncture, compared with conventional treatment, is beneficial to migraine by reducing consumption of medication [13,14]. Li *et al.* discovered that stimulation of genuine acupoints was superior to stimulation of non-acupoints in relieving pain and preventing migraine relapse or aggravation [15]. Moreover, a review of clinical trials revealed that acupuncture is an effective treatment option for migraine prophylaxis [16].

In this study, patients with migraine were used as subjects to investigate acupoint specificity. We used fluorodeoxyglucose positron emission tomography combined with computed tomography (FDG-PET/CT). FDG-PET is used to visualize glucose metabolism and is frequently applied for diagnosis of various diseases. In recent years, FDG-PET has also been used to assess brain function related to the efficacy of acupuncture [17]. Patients received stimulation at specific acupoints of the Shaoyang meridians, which are traditionally employed in the treatment of migraine. In the control group, acupoints on the Yangming meridians were stimulated. These points are less often used than points on Shaoyang meiridians for migraine treatment according to clinical data and theories of traditional acupuncture. We hypothesized that the specific and non-specific stimulation would elicit distinct patterns of brain activity. This could provide information on the specificity of acupoints in the treatment of migraine.

## Methods

### Subjects and experimental paradigm

Thirty right-handed patients with acute migraine without aura, selected from a total of 278 patients recruited from July 2008 to September 2009, were studied. The migraine patients (12 males and 18 females; mean age 32.87 ± 8.71 years) were randomized into three groups: 1) Traditional Acupuncture Group (TAG), 2) Control Acupuncture Group (CAG), and 3) Migraine Group (MG). The TAG received specific stimulation of traditional acupoints, the CAG received non-specific stimulation, and the MG received no treatment. Moreover, after PET-CT scan, MG group were given a fee for their contribution, and also we would give them acupuncture treatment for free if they want. Subjects were matched by gender, age, handedness, and education. Each subject gave informed consent and all study protocols were approved by the ethics committee of the Affiliated Hospital of Chengdu University of Traditional Chinese Medicine.

The inclusion criteria were: (1) meeting the classification criteria of the International Headache Society for the diagnosis of migraine without aura; (2) left-sided headache; (3) one or more migraine attacks per month during the last three months; (4) a Visual Analogue Scale (VAS) score of 2–8 at recruitment and before scanning; (5) less than 24 hours from the previous migraine attack to the beginning of the scan; (6) age 20–45 years; (7) negative neurological examination and normal skull CT or MRI; (8) no medication for migraine within 24 hours since the onset of the acute attack; and (9) being capable of giving written informed consent.

The exclusion criteria were: (1) headaches caused by organic disorders, such as subarachnoid hemorrhage, cerebral hemorrhage, cerebral embolism, cerebral thrombosis, vascular malformation, hypertension, or arteriosclerosis; (2) presence of psychosis, bleeding disorders, or allergies that may preclude the safe use of acupuncture; (3) presence of concurrent autoimmune or inflammatory disease resulting in pain; (4) concurrent participation in other studies; (5) pregnancy or nursing; (6) medication with vasoactive agent in the last two weeks; (7) current major anxiety disorder and/or depression; and (8) presence of any contraindications to PET-CT or electroacupuncture.

**Figure 1:**
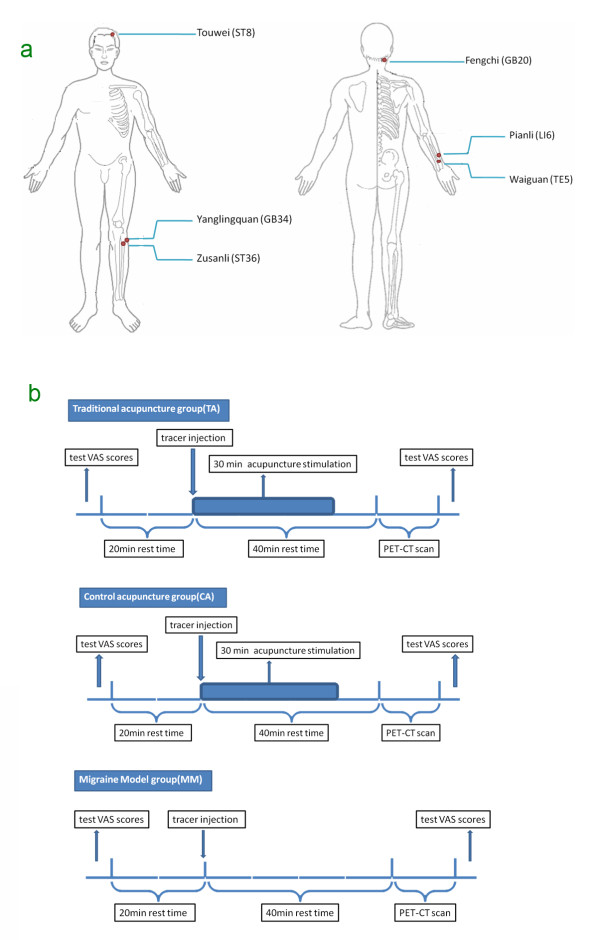
** a: Location of acupoints. b:** Experimental Paradigm.

Acupuncture treatment was applied to the TAG and CAG by electroacupuncture treatment (EAT), while the MG was not treated in any way. Acupuncture stimulation (AS) in the TAG was performed at Waiguan (TE5), Yang Lingquan (GB34), and Fengchi (GB20) on the Shaoyang meridians. In the CAG, AS was performed at Touwei (ST8), Pianli (LI6), and Zusanli (ST36) on the Yangming meridians (Figure 1a). Sterile single-use acupuncture needles (25–40 mm in length and 0.30 mm in diameter; manufactured by Suzhou Medical Supplies Co., Ltd., Suzhou, China) were used for stimulation. The treated subjects achieved DeQi sensation (soreness, numbness, distention, and heaviness) by the manipulations of lifting and thrusting or twirling and rotating. Once all the acupoints had been needled, auxiliary needles were perpendicularly punctured 2 mm lateral to the acupoints, to 2 mm in depth, without manual manipulation. Electro-acupuncture (HANS: Han's acupoint nerve stimulator, HANS-200, Nanjing, China) was performed on the acupoints by one experienced acupuncturist. Each acupuncture needle and its auxiliary needle were connected with the electricity by HANS for 30 minutes. The stimulation frequency was 2/100 Hz, and the stimulation intensity varied from 0.1 to 1.0 mA as long as the patients felt comfortable, as determined by the previous studies [15,18].

PET-CT scans were performed on the subjects at the PET-CT center of the Sichuan Provincial People’s Hospital. When the migraine attack began, each subject went through the following procedure: (1) examinations of blood sugar and Visual Analogue Scale (VAS) scores (range from 0 to 10) before the PET-CT scan; (2) a 20 min rest in a quiet room; (3) a tracer injection at the back of the right hand (18F-FDG; synthesized with Mini Tracer accelerator; 0.11 mCi/kg dosage); (4) a 40 min rest, which included the 30 min acupuncture treatment in the TAG and CAG; (5) a PET-CT scan; and (6) examination of VAS scores after the PET-CT scan (Figure 1b). Subjects were instructed to remain relaxed during the whole study, with eyes blindfolded and ears plugged.

### PET-CT imaging

It has been found that curative effects of acupuncture treatment emerge after about 30 to 40 minutes of acupuncture stimulation [15,18], and 18F-FDG is known to be stable in the brain for 30 to 45 minutes after injection (half-life ~109 min). We therefore acquired image data sets at 40 minutes after the 18F-FDG injection, with the aim of capturing the processes underlying the effect of acupuncture.

PET-CT scans were performed using a Biograph Duo BGO scanner (Siemens, Germany). The images covered the whole brain and were parallel to the AC-PC line. Image acquisition was started after a 40 min uptake period (bed: 1; collection mode: 3D; slice thickness: 3 mm; slice interval: 1.5 mm; matrix size: 256 × 256; total counts: 3 × 109). On completion of data acquisition, the images were reconstructed using ordered-subset expectation maximization (OSEM) with 6 iterations and 16 subsets.

### Image processing

PET-CT images were processed using SPM2 software (Wellcome Department of Cognitive Neurology, University College London, UK). After realignment, the images were normalized using the Montreal Neurological Institute (MNI) template and then smoothed using a Gaussian kernel with 6 mm full width at half maximum (FWHM). On the second level of statistical analysis, the differences between the TAG and MG and between the CAG and MG were tested using separate two-sample t-tests. The statistical threshold was set at P <0.05 with correction for false-discovery rate (FDR) and the cluster size threshold was >5 voxels.

## Results

### Effect of acupuncture on pain

**Figure 2:**
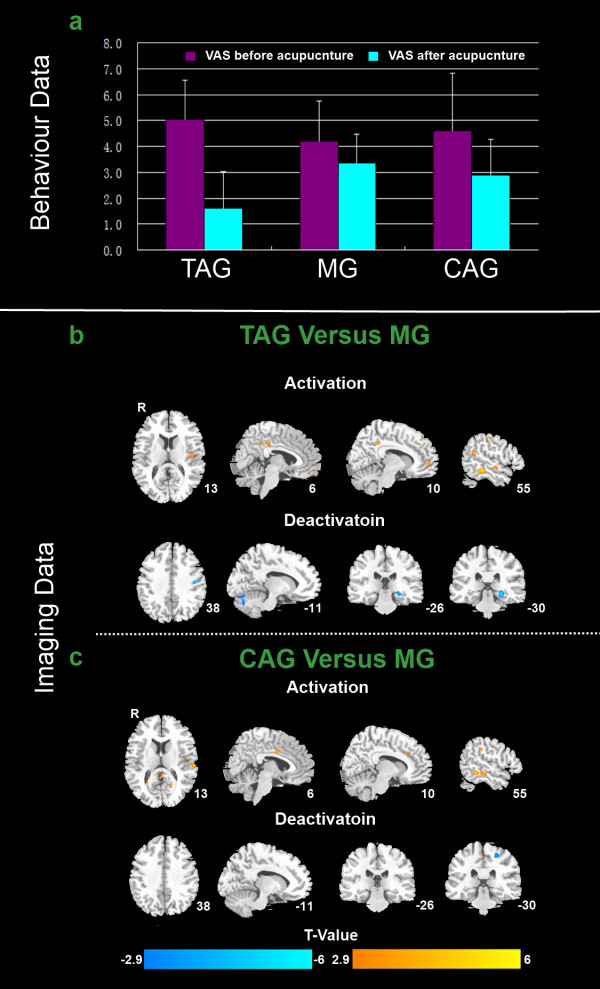
** a: Behavior data analysis. b** and **c**: Imaging data analysis.

The VAS of pain intensity was significantly reduced in the TAG (P = 0.0005) and CAG (P = 0.008) groups after AS compared with before (paired two-tailed *t*-test with threshold at P <0.01). The reduction of pain intensity appeared greater in the TAG than in the CAG. There was no significant reduction in pain intensity in the MG (P = 0.047) (Figure 2a).

## PET results

In the TAG, metabolism increased compared with the MG in the middle temporal cortex (MTC), orbital frontal cortex (OFC), insula, middle frontal gyrus, angular gyrus, posterior cingulate cortex (PCC), precuneus, and middle cingulate cortex (MCC). Metabolism decreased in the parahippocampus, hippocampus, fusiform gyrus, postcentral gyrus, and cerebellum (Figure 2b and Table 1). In the CAG, metabolism increased compared with the MG in the MTC, supratemporal gyrus, supramarginal gyrus, and MCC, whereas metabolism decreased in the cerebellum (Figure 2c and Table 1).

**Table 1:** **Main localization of activated/deactivated brain regions, comparing SG or MSG versus MG, using two-sample*t*-test**

**TAG-MG**								**CAG-MG**					
**Regions**	**Hem**	**BA**	**Talairach**			**T-score**	**Vol**	**BA**	**Talairach**			**T-score**	**Vol**
			**x**	**y**	**z**				**x**	**y**	**z**		
Medial Frontal	L	9/10	−10	−50	−4	3.97	16						
	R												
Middle	L	6/9	−30	36	28	3.48	17						
Frontal	R												
Middle	L	17	−59	−29	7	3.89	17	21	−57	−26	−9	4.43	23
Temporal	R												
Postcentral	L	2	−61	−22	32	−2.98	6						
Gyrus	R												
Fusiform	L	18/19	−28	−86	−18	−3.36	6						
	R	18/19	22	−82	−14	−3.06	6		33	−48	−35	−3.58	27
Precuneus	L	31/39	−40	−68	37	3.73	11						
	R	31/39	12	−51	32	4.82	14						
ACC	L	32	−12	48	−4	3.72	6						
	R												
MCC	L	31	−2	−36	26	2.99	65	31	−10	31	28	3.56	18
	R	31	8	−55	29	3.02	20						
PCC	L	23/31	−2	−38	24	3.05	6						
	R	23/31	6	−38	24	3.07	24						
Insula	L	13	−44	−9	13	3.61	21						
	R												
Hippocampus	L		−28	−28	−7	−4.75	5						
	R												
Parahippocampus	L	19	−30	−49	−4	−3.50	5						
	R												
Cerebellum	L		−28	−84	−18	−3.30	10		−30	−82	−16	−3.19	23
	R		6	−68	−8	−4.31	200		4	−68	−7	−3.43	94

**Abbreviations:** BA-Brodmann area; Hem-hemisphere; L-Left; R-Right; ACC-anterior cingulate cortex; MCC-middle cingulate cortex ; PCC-poster cingulate cortex.

## Discussion

According to the theories of acupuncture and TCM, acupoints are reactive sites of diseases on the body surface, with close relationships to specific visceral organs and meridians. In this study, we applied PET neuroimaging technology to investigate the concept of acupoint specificity in migraine patients. The results showed that analgesia was more effective in the TAG than in the CAG, and that the two groups showed cerebral patterns of metabolism that were distinct from the MG.

In both groups receiving AS, there was an increase in metabolism in the MTC and MCC, and a decrease in metabolism in the cerebellum, compared with the MG. The MTC is located in the temporal lobe, but its exact function is still unknown. Functional neuroimaging studies have indicated that the MTC is concerned with cognitive processes such as language and semantic memory processing, and multimodal sensory integration [19,20]. We suggest that MTC may be related to the reaction of the body to the external AS. The MCC is a part of the limbic system, which is an important region in acute pain and anxiety [21,22]. Brown *et al.* found that the MCC could play a role in interrupting attention during the anticipation of pain [1]. Moreover, MCC is implicated in pain, emotion and cognition. Some investigator reported that MCC might be an important site for the interaction between negative emotion and motor signals [23]. We hypothesize that acupuncture—specific or non-specific—might modulate certain components of the pain matrix. However, the question of whether the change in brain activity induced by acupuncture is related to self-regulation and/or emotional factor requires further investigation. The human cerebellum is a complicated region. It is well-known to play an important role in motor control, but it also has a crucial role in affective behaviors such as responses to fear and pleasure [1]. Previous animal study indicated that the cerebellum contributes more to pain processing than just motor control [24]. Our results indicate that the cerebellum may be affected by acupuncture. We speculated that cerebellar metabolism might be related to nociceptive processing or motor preparation. The increase in metabolism in the MTC and MCC was greater in the CAG than in that TAG, indicating that the non-specific AS might require more sensory integration or attention.

Metabolism changes were observed in the MFC, OFC, insula, PCC, precuneus, parahippocampus, and hippocampus in the TAG but not in the CAG. Recently, an [11C]-PET study suggested that the OFC may be involved in endogenous opioid modulation during acupuncture analgesia [4]. The insula receives input from the spinothalamically activated posterior thalamic nuclei, and has been shown to be involved in the processing of visceral sensory input, attention, pain, emotion, and visual input [25]. The insula has been consistently been found to be activated in experimental pain studies. It has links with the limbic and autonomic systems and is thought to be involved in the representation of the emotional aspect of pain. A PET study of patients with spontaneous migraine reported activation of the insula [26]. This was confirmed in our study. The hippocampus is a major component of the human brain that links affective states with memory processing, and that has a role in pain processing [27]. The greater changes seen in the TAG compared with the CAG in the regions discussed above—in particular the OFC, insula, parahippocampus, and hippocampus—are consistent with their close relationship to pain processing. Our findings indicate that the limbic system is central to the effect of acupuncture in migraine patients. We speculate that stimulation of the traditional acupoints, which are used clinically for migraine treatment, may deactivate brain regions associated with migraine or pain. The different patterns of metabolism observed could reflect acupoint specificity under pathogenic conditions.

## Conclusions

We have reported that acupuncture stimulation of different points on similar body regions in migraine patients reduced pain and induced different patterns of cerebral glucose metabolism in the brain. Our findings demonstrated that acupoints did have some specificity in migraine treatment. Further studies using patients with different pathological conditions should be performed to study the specificity of acupoints.

## Competing interests

No competing interests exist.

## Authors’ contributions

Study protocol and design: FL, FZ, JY. Acquisition of data: JY, FZ, YF, LF, WQ, XL, WS, HX. Analysis and interpretation of data: WQ, WS, HX. Drafting of the manuscript: JY, FZ, YF, WQ, JC. Revision of the manuscript: FL, JY, FZ, YF. All authors read and approved the final manuscript.

## Pre-publication history

The pre-publication history for this paper can be accessed here:

http://www.biomedcentral.com/1472-6882/12/123/prepub
